# Monocyte accumulation is an early event in HAM/TSP pathogenesis, while monocyte activation and IFN-regulated gene expression persist in chronic HAM/TSP

**DOI:** 10.1186/1742-4690-12-S1-O25

**Published:** 2015-08-28

**Authors:** Soraya Maria Menezes, Harry Freitag Muhammad, George Soares, Ricardo Khouri, Daniele Decanine, Gilvaneia Silva Santos, Saul Velloso Schnitman, Ramon Kruschewsky, Giovanni López, Carolina Alvarez, Michael Talledo, Eduardo Gotuzzo, Bernardo Galvão-Castro, Johan Van Weyenbergh

**Affiliations:** 1 Rega Institute for Medical Research, Department of Microbiology and Immunology K.U, Leuven, Belgium; 2 LIMI-CPqGM, Oswaldo Cruz Foundation (FIOCRUZ), Salvador-Bahia, Brazil; 3 Bahiana School of Medicine and Public Health, Salvador-Bahia, Brazil; 4 Instituto de Medicina Tropical Alexander von Humboldt, Universidad Peruana Cayetano Heredia, Lima, Peru; 5 Departamento de Medicina, Facultad de Medicina Alberto Hurtado, Universidad Peruana Cayetano Heredia, Lima, Peru

## 

Tattermusch et al (2012) identified an IFN-inducible gene signature in whole blood of HAM/TSP patients, with a strong myeloid component, while abortive HTLV-1 infection induces monocyte apoptosis (Sze et al. 2013). We previously demonstrated that B cell CD80 expression correlates to disease severity in HAM/TSP (Menezes et al 2014), whereas B cell CD86 is selectively up-regulated by IFN-beta in both HAM/TSP and multiple sclerosis (MS). In this study, we propose a cell type-and gene-specific, rather than a generalized IFN response in HAM/TSP. Using polychromatic flow cytometry, comprehensive phenotyping of monocytes (CD14, CD64, CD80, CD86, CD95/Fas, HLA-DR) was performed in a total of 53 individuals (HAM/TSP patients, asymptomatic HTLV-1-infected and uninfected controls), and absolute and relative monocyte counts were obtained from >600 HTLV-1-infected individuals with complete clinical follow-up and proviral load. Ex vivo monocyte levels increased in early HAM/TSP (p<0.01), independent of proviral load, and were significantly correlated to age of onset of HAM/TSP in both Brazilian and Peruvian cohorts. On the other hand, monocyte activation measured by systemic soluble CD14 was significantly increased in chronic (p<0.01) but not early HAM/TSP, whereas CD95 and CD86 expression in monocytes correlated negatively to disease progression. Interestingly, membrane expression of CD14 is down-regulated and CD95/CD86 up-regulated by IFN-beta in vitro (controls) and in vivo (MS), suggesting IFN-regulated expression of all three monocyte receptors in HAM/TSP. Transcriptomic analysis of whole blood vs. purified monocytes/B cells confirmed cell-specific expression of CD64/CD80/CD86 ex vivo, whereas a selective decrease of myeloid/monocyte-specific genes was observed upon in vitro culture of HAM/TSP PBMCs, possibly due to apoptosis of specific monocyte subsets. In conclusion, an increase in soluble CD14, as well as monocyte-specific expression of CD64, CD95 and CD86 differentially reflect disease progression in HAM/TSP, in addition to B-cell specific CD80 expression, arguing for a complex and compartmentalized IFN response.

